# Longitudinal associations between psychological well-being and depressive symptoms among older adults in the Baltic states

**DOI:** 10.3389/fpsyg.2025.1649231

**Published:** 2025-10-02

**Authors:** Antanas Kairys, Vytautas Jurkuvėnas, Iluta Skrūzkalne, Vita Mikuličiūtė, Diāna Kalniņa, Olga Zamalijeva

**Affiliations:** ^1^Statistics Unit, Rīga Stradiņš University, Riga, Latvia; ^2^Institute of Psychology, Faculty of Philosophy, Vilnius University, Vilnius, Lithuania

**Keywords:** psychological well-being, depressive symptoms, ageing, longitudinal study, Baltic states

## Abstract

Depression in later life is a pressing public health concern that is often comorbid with chronic illness and associated with substantial declines in psychological well-being. Drawing on the dual continua model of mental health, this study investigated the longitudinal, bidirectional associations between psychological well-being and depressive symptoms among older adults in the Baltic States. Using two waves of data (2019/2020 and 2021/2022) from the Survey of Health, Ageing and Retirement in Europe (SHARE), we analyzed responses from 5,874 individuals aged 50 and above in Estonia, Latvia, and Lithuania. Psychological well-being was assessed using the CASP-12 scale, and depressive symptoms using the EURO-D scale. A cross-lagged panel model, adjusted for age, gender, and multimorbidity, revealed that both constructs were moderately stable over time and negatively associated in each wave. Notably, psychological well-being at baseline significantly predicted depressive symptoms 2 years later (*β* = −0.17, *p* < 0.001), and depressive symptoms also predicted subsequent well-being (*β* = −0.07, *p* < 0.001), suggesting a bidirectional relationship with stronger effects from well-being to depression. These findings support the dual continua model and underscore the importance of promoting psychological well-being to mitigate depression risk in ageing populations.

## Introduction

Depression in later life represents a growing public health concern, posing significant challenges to both individuals and society as a whole (Paun, 2023). Given the demographic shift toward larger older adult populations, understanding the mechanisms underlying the onset and progression of late-life depression is a key public health priority. Depressive symptoms in old age are associated not only with heightened risks of cardiovascular disease and cancer-related mortality (van Zutphen et al., 2021; Wang et al., 2020), but also with accelerated declines in self-rated mobility and daily functioning (Yang et al., 2021). Furthermore, good mental health in older age is crucial for maintaining interpersonal relationships, supporting community engagement, and sustaining economic participation (Curran et al., 2020).

Prevalence estimates highlight the urgency of addressing late-life depression: a meta-analysis by Hu et al. (2022) reports a global prevalence of approximately 28.4% among older adults, though estimates vary widely depending on diagnostic criteria and regional context, while it is estimated that up to half of clinical depression cases remain undiagnosed (Zenebe et al., 2021). Depression in this population not only exacerbates existing health problems but also increases interpersonal strain, functional impairment, treatment complications, and, in severe cases, risk of suicide (Alexopoulos, 2005; Fiske et al., 2009).

A wide range of factors contribute to the onset of depression in older adulthood, including genetic predispositions, age-related cognitive and neurobiological decline, and adverse life events such as bereavement or social isolation (Fiske et al., 2009). Additionally, gender differences are consistently observed, with women reporting higher levels of depressive symptoms than men (Labaka et al., 2018), and older adults exhibiting higher prevalence rates than younger individuals (Zhao et al., 2012). Chronic health conditions and physical disability further elevate the risk (Alexopoulos, 2005; Stickle and Onedera, 2006). Multimorbidity, in particular, has been linked to reduced psychological well-being and an elevated risk of depression (Makovski et al., 2019; Read et al., 2017). These findings underscore the need to account for age, gender, and health status in research on late-life depression.

The relationship between psychological well-being and depression has traditionally been viewed as a continuum, where well-being and depressiveness are conceptualized as opposite ends of the same scale (Siddaway et al., 2017). This assumption is reflected in measurement tools such as the Psychological General Well-Being Index, which incorporates both positive affect and depressive symptoms (Gaston and Vogl, 2005). However, this unidimensional view has been challenged by the dual continua model, which posits that mental health and mental illness represent distinct, but interrelated constructs (Westerhof and Keyes, 2009). This framework has received growing empirical support (Headey et al., 1993; Huppert and Whittington, 2003; Grant et al., 2013; Wood and Joseph, 2010), suggesting that low psychological well-being may not simply be the symptom of mental illness, but may serve as a precursor to it. However, it has been seldom studied in older adult populations and has predominantly focused on English-speaking countries such as the United Kingdom (Huppert and Whittington, 2003), the United States (Grant et al., 2013; Wood and Joseph, 2010), and Australia (Headey et al., 1993).

### Objective

The aim of this study is to evaluate the longitudinal associations between psychological well-being and depressive symptoms using two waves of data from the Survey of Health, Ageing and Retirement in Europe (SHARE) collected in Estonia, Latvia, and Lithuania.

## Method

### Sample characteristics

This analysis utilized data from Wave 8 (2019/2020) and Wave 9 (2021/2022) of the Survey of Health, Ageing and Retirement in Europe (SHARE) for Estonia, Latvia, and Lithuania (Börsch-Supan et al., 2013; SHARE-ERIC, 2024a; SHARE-ERIC, 2024b). The sample consisted of *N* = 5,874 adults aged 50 and older who participated in at least one of the two waves and had data on psychological well-being or depressive symptoms. The decision to study individuals aged 50 and older was based on the fact that the age of 50 is a conventional threshold in ageing research (e.g., ELSA, SHARE). Although 50-year-olds are not formally considered old, they already encounter challenges characteristic of older adults, such as ageism (Allen et al., 2022). The three national samples were pooled, as these Baltic countries have comparable cultural and socio-economic profiles, allowing for a combined analysis. The Wave 8 (baseline) interview age ranged from 50 to 100 years (M = 69.1, SD = 10.4). Women comprised 62.9% of the sample (*n* = 3,659). About 46.6% of respondents had zero or one chronic health condition, whereas 53.2% reported presence of multimorbidity, defined as two or more chronic diseases. 58.1% of participants lived with partner in household, 28.4% had a tertiary education degree. By country, 55.8% of participants were from Estonia (*n* = 3,279), 24.0% from Lithuania (*n* = 1,411), and 20.2% from Latvia (*n* = 1,185).

### Primary outcomes

Psychological well-being was measured using the 12-item version of the Control, Autonomy, Self-Realisation and Pleasure scale (CASP-12; Hyde et al., 2003; von dem Knesebeck et al., 2005). The items assess perceived control, autonomy, enjoyment of life, and future outlook among older adults (e.g., “How often do you feel that life is full of opportunities?”). Each item is rated on a four-point Likert scale, ranging from 1 (“never”) to 4 (“often”). A total score was used in the analysis, with higher scores indicating greater psychological well-being. Previous research supports the scale’s psychometric soundness, including good reliability and justification for using the total score (Kerry, 2018). In this study Cronbach’s alpha was 0.81 (Wave 8) and 0.80 (Wave 9).

Depressive symptoms were assessed using the EURO-D scale (Prince et al., 1999), a widely validated self-report instrument for measuring depression in older adults. The scale includes items related to sadness, loss of interest, suicidality, self-blame, sleep disturbances, appetite changes, irritability, concentration difficulties, and other depression-related symptoms (Maskileyson et al., 2021). Each item is scored dichotomously (1 = symptom present, 0 = symptom absent), and the total score was used in the analysis, with higher scores reflecting more severe depressive symptoms. The scale has demonstrated good internal consistency and supports the use of a general factor score in older populations (Tomás et al., 2022). In this study Cronbach’s alpha was 0.70 (Wave 8) and 0.69 (Wave 9).

Control variables included age, gender, and the number of chronic diseases. Based on the most common definition, multimorbidity was operationalized as a binary variable indicating whether the respondent reported having two or more chronic conditions (Johnston et al., 2019). Control variable data was derived from Wave 8.

### Statistical analysis

The data analysis focused on a cross-lagged panel model (CLPM) that was specified using the R package lavaan (version 0.6.17; Rosseel, 2012), applying the robust maximum likelihood (MLR) estimator. Full information maximum likelihood (FIML) estimation was employed to handle missing data under the assumption of missing at random (MAR). All variables, including both, depressive symptoms and psychological well-being level in Wave 8 and Wave 9, as well as control variables (age, gender, presence of multimorbidity), were modelled as observed variables. The cross-lagged structure included autoregressive and cross-lagged paths to examine reciprocal effects over time. Model fit was evaluated using multiple fit indices: the Root Mean Square Error of Approximation (RMSEA), Comparative Fit Index (CFI), and Tucker–Lewis Index (TLI). Following established guidelines, good model fit was defined as RMSEA <0.06 (acceptable <0.08), and CFI and TLI ≥0.95 (Hu and Bentler, 1999; Marsh et al., 2004). Statistical significance was evaluated at *p* ≤ 0.05.

## Results

The means, standard deviations, and Pearson correlations between age, gender, multimorbidity, psychological well-being (Waves 8 and 9), and depressive symptoms (Waves 8 and 9) are presented in Table 1. All constructs showed significant correlations, except for the non-significant relationship between gender and psychological well-being.

**Table 1:** Means, standard deviations, and Pearson correlations between study variables (*N* = 5,874).

Variable	M	SD	1	2	3	4	5	6	7
1. Gender (0 = male)	0.63	0.48	—						
2. Age	69.07	10.45	0.09^***^	—					
3. Multimorbidity (0 = no)	0.53	0.50	0.08^***^	0.29^***^	—				
4. Psychological well-being (Wave 8)	35.5	6.37	−0.01	−0.28^***^	−0.30^***^	—			
5. Psychological well-being (Wave 9)	35.3	6.23	0.00	−0.27^***^	−0.26^***^	0.67^***^	—		
6. Depressive symptoms (Wave 8)	2.88	2.30	0.15^***^	0.21^***^	0.30^***^	−0.56^***^	−0.40^***^	—	
7. Depressive symptoms (Wave 9)	2.90	2.29	0.15^***^	0.20^***^	0.23^***^	−0.42^***^	−0.52^***^	0.56^***^	—

^***^p < 0.001.

**Figure 1:**
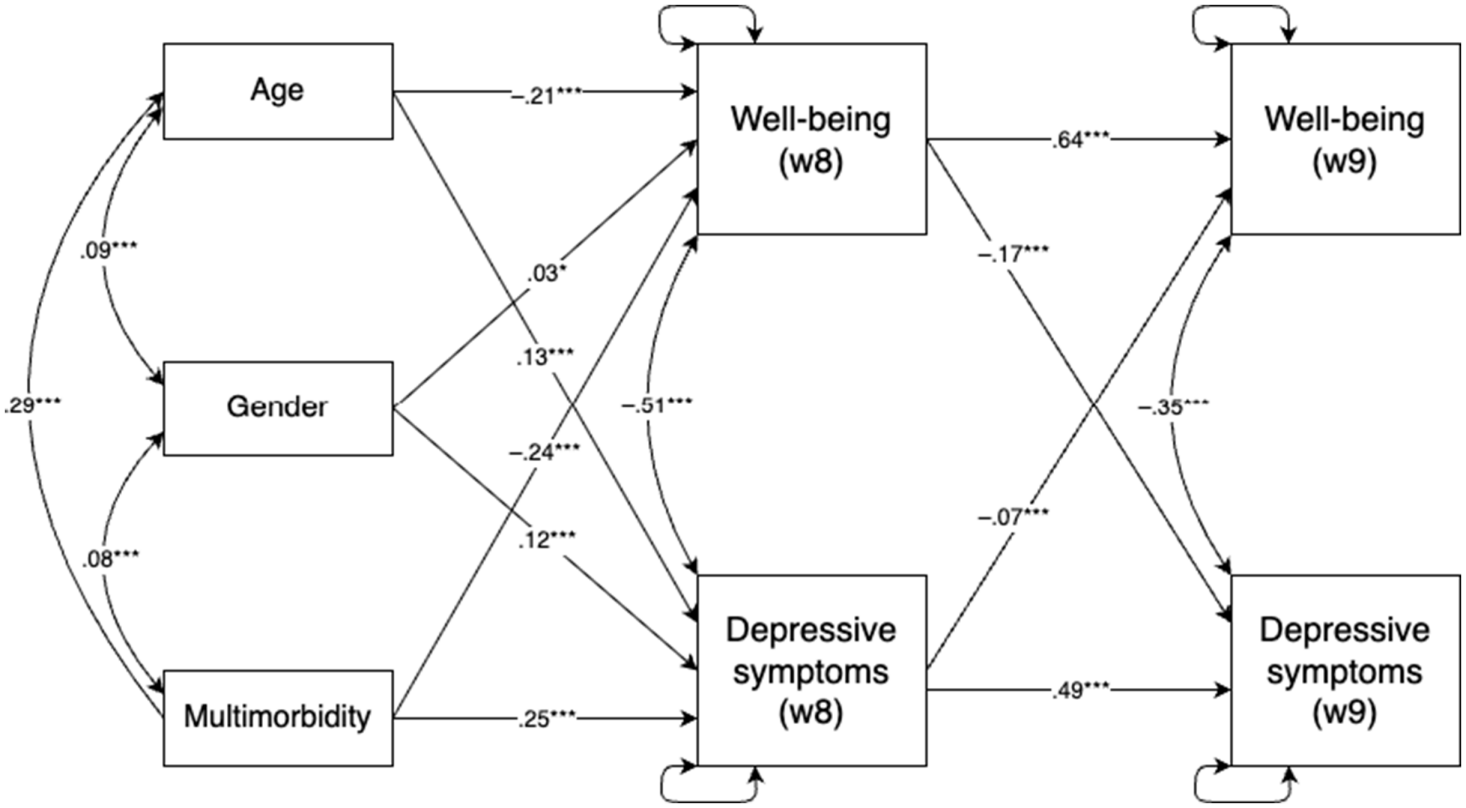
Autoregressive cross-lagged panel model examining the bidirectional associations between psychological well-being and depressive symptoms across two time points (*N* = 5,874). ^*^p < 0.001 and ^***^p < 0.001.

The cross-lagged model demonstrated an adequate data fit, *χ*^2^(6) = 177.71, *p* < 0.001; CFI = 0.98; TLI = 0.94; RMSEA = 0.07, 90% CI (0.061, 0.079). Examination of the standardized coefficients (see Figure 1) indicated acceptable stability for both psychological well-being and depressive symptoms over the two-year period, with psychological well-being demonstrating slightly higher longitudinal stability.

The concurrent correlations between psychological well-being and depressive symptoms were also substantial. All control variables (age, gender, and multimorbidity) were significantly associated with both well-being and depressive symptoms in Wave 8. Specifically, age and multimorbidity were negatively associated with psychological well-being and positively with depressive symptoms, whereas gender showed positive associations with both outcomes.

Regarding cross-lagged effects, controlling for autoregressive paths and covariates, psychological well-being in Wave 8 significantly predicted depressive symptoms in Wave 9 (*β* = −0.17), and depressive symptoms in Wave 8 predicted well-being in Wave 9 (*β* = −0.07). Both effects were negative, with the path from psychological well-being to later depressive symptoms being slightly stronger.

## Discussion

The present study focused on examining the associations between psychological well-being and depressiveness among older adults using data from the Survey of Health, Ageing, and Retirement in Europe collected in the Baltic states. By employing a cross-lagged panel model, the longitudinal relationships between these constructs were explored while controlling for key factors such as age, gender, and multimorbidity. The findings offer valuable insights into the complex relationship between psychological well-being and depressive symptoms, adding to the growing body of evidence supporting the dual continua model of mental health (Iasiello et al., 2020) in a less researched population.

### Key findings and interpretations

First, the results demonstrated substantial consistency for both psychological well-being and depressive symptoms across the two-year period, with well-being showing slightly higher rank-order stability. This finding is consistent with prior research suggesting that psychological well-being is a relatively enduring construct not only in the short but also in the long term shaped by stable personality traits and life circumstances (Huppert and Whittington, 2003; Diener et al., 2017). It also aligns with the proposition of set point theory, which posits that individuals tend to maintain a relatively stable level of well-being. In other words, after various life events, people typically return to their baseline level of well-being. The contemporary view of set point theory acknowledges that majority of adults in Western countries maintain stable levels of life satisfaction due to stabilizing factors such as dispositional traits and early parental influences—a view sometimes described as a “weak” version of set-point theory (Headey et al., 2014). However, it is important to note that the findings of this study should be interpreted with caution, as they capture only short-term consistency rather than long-term stability, and research covering longer periods would provide a better understanding of well-being stability. Depressive symptoms likewise demonstrate moderate stability over time (Burns et al., 2013), although they tend to fluctuate more than psychological well-being in response to acute life stressors and situational changes. This difference in stability may be explained by the greater reactivity of depressive symptoms to short-term environmental and psychological stressors (Wetherell et al., 2001). Accordingly, late-life depression has been shown to be particularly sensitive to factors such as health decline (Power et al., 2017), bereavement (Boerner et al., 2024), and changes in social support system (Chen, 2020), highlighting the dynamic nature of mental health in older adulthood. In our study, the lower consistency of depressive symptoms may partly reflect the timing of data collection, as the interval between assessments coincided with the COVID-19 pandemic.

The associations observed between psychological well-being and depressive symptoms at each measurement point were consistent with the dual continua model, which continues to gain empirical support (Iasiello et al., 2020). Although negative, these associations underscore that psychological well-being and depressive symptoms are related yet distinct constructs. This challenges the traditional view that they lie on a single continuum and reinforces previous research emphasizing the importance of addressing both mental health and mental illness as separate, though interconnected, domains (Westerhof and Keyes, 2009).

Importantly, the cross-lagged analysis revealed bidirectional influences between psychological well-being and depressive symptoms, with well-being at the first assessment significantly predicting depressive symptoms at the second assessment, and vice versa. The stronger influence of psychological well-being on subsequent depressive symptoms, compared to the reverse, aligns with previous findings suggesting that lower levels of well-being can serve as a precursor to the onset of depressive symptoms over time (Grant et al., 2013; Wood and Joseph, 2010). This highlights the critical role of psychological well-being in mitigating the risk of depression, even in older populations with heightened vulnerability due to age-related stressors and health challenges.

### Implications for research and practice

These findings highlight the importance of adopting a dual-focus approach in both research and clinical practice when addressing mental health in older adults. Conceptualizing psychological well-being and depressive symptoms as distinct yet interconnected constructs offers a more nuanced framework for understanding the complexity of mental health in this population (Westerhof and Keyes, 2009). Other recent longitudinal research confirms that low levels of well-being are a risk factor for future depression and that improving psychological well-being may help reduce depressive symptoms over time (Joshanloo and Blasco-Belled, 2023). Interventions aimed at enhancing psychological well-being, such as promoting social engagement, fostering a sense of purpose, and supporting the management of chronic health conditions, may therefore not only improve quality of life but also contribute to the prevention or mitigation of depression (Restrepo-Escudero et al., 2024).

Moreover, the observed bidirectional relationship suggests a potential reinforcing cycle: declines in psychological well-being may exacerbate depressive symptoms, which in turn may further erode well-being. This underscores the need for early detection and timely interventions to break this cycle and foster psychological resilience in older populations.

From a clinical standpoint, these findings support the inclusion of psychological well-being assessments alongside traditional depression screening. Given that low well-being can precede depressive episodes, incorporating positive mental health indicators into clinical evaluations could enable earlier identification of at-risk individuals and support more holistic and preventative treatment strategies (Franken et al., 2018).

### Limitations and future directions

While this study offers valuable evidence on the relationship between psychological well-being and depressive symptoms, several limitations should be acknowledged. First, the generalizability of the findings is constrained to the context of the Baltic countries and may not extend to populations with different cultural or socio-economic backgrounds. Although, as noted earlier in the discussion, some of the findings were consistent with those reported in the United States (Grant et al., 2013; Wood and Joseph, 2010) and the Netherlands (Westerhof and Keyes, 2009), it is important to recognize that both depressive symptoms and psychological well-being are sensitive to environmental stressors and cultural factors (Wetherell et al., 2001; Wirtz et al., 2009). Consequently, it cannot be excluded that testing the full model in a different cultural context might produce different results. Future studies should aim to replicate these findings across more diverse samples to examine potential cross-cultural variations in the dynamics between psychological well-being and depressive symptoms.

Second, although the longitudinal design allows for stronger inferences regarding temporal associations, the study was based on only two measurement points, as only two points are available for Latvia and Lithuania. Moreover, the period between the first and second measurement coincided with the COVID-19 pandemic, which may also have biased the results. This limits the ability to examine long-term trajectories and reduces the robustness of conclusions about lasting changes or sustained bidirectional effects. The two time points also limited the possibility of applying models other than the classical cross-lagged model, such as the random intercept cross-lagged model, since these require three or more measurement points. Future research employing multiple time points would be better suited to capture complex developmental patterns and test for sustained reciprocal effects.

Third, while self-report instruments are validated and commonly used in psychological research, they remain susceptible to recall bias, social desirability effects, and other forms of reporting error. Supplementing self-report data with objective indicators or clinician-administered assessments could enrich our understanding of the well-being-depression relationship.

Finally, the correlational nature of the study means that causality cannot be definitively established. Experimental or intervention-based studies are needed to directly test whether promoting psychological well-being can causally reduce depressive symptoms over time.

## Conclusion

This study contributes to the growing evidence supporting the dual continua model of mental health, emphasizing the distinct yet interconnected nature of psychological well-being and depressive symptoms. By demonstrating the bidirectional relationship between these constructs, it highlights the importance of promoting psychological well-being as a means of reducing depressive symptoms and improving overall mental health in older adults. Given the ageing population and the associated burden of health challenges, these findings have important implications for public health strategies, clinical practices, and future research aiming to enhance the quality of life in later years.

## Data Availability

Publicly available datasets were analyzed in this study. This data can be found here: https://share-eric.eu/data/data-access.
